# BMI, Blood Pressure, and Plasma Lipids among Centenarians and Their Offspring

**DOI:** 10.1155/2022/3836247

**Published:** 2022-01-20

**Authors:** Peng-Cheng Hai, Dong-Xiao Yao, Rui Zhao, Chen Dong, Sara Saymuah, Yue-Song Pan, Zhi-Feng Gu, Yi-Long Wang, Chen Wang, Jian-Lin Gao

**Affiliations:** ^1^School of General Practice and Continuing Education, Capital Medical University, Fengtai, No. 10, You An Men Wai Xi Tou Tiao, Beijing 100069, China; ^2^Department of Neurology, Beijing Tiantan Hospital, Capital Medical University, Beijing, China; ^3^Research Center of Gerontology and Longevity, Affiliated Hospital of Nantong University, Nantong, China; ^4^Department of Neurosurgery, Wayne State University School of Medicine, Detroit, USA; ^5^Department of General Practice, Beijing Tiantan Hospital, Capital Medical University, Beijing, China

## Abstract

**Background:**

The burden of cardiovascular diseases (CVDs) is increasing substantially due to population growth and aging. Determining effective prevention and understanding the underlying mechanisms remain desirable pursuits for increasing the quality of life. As centenarians and their offspring may have genetic advantages, they may present with healthier cardiovascular-related profiles.

**Methods:**

We launched a cross-sectional household-based survey of centenarian families, including 253 centenarians, 217 centenarian offspring, and 116 offspring spouses without centenarian parents from county-level Chinese longevity city Rugao. Among offspring and offspring spouses were the following arrangements: 101 paired offspring and offspring spouses who lived together, 116 unpaired offspring, and 16 unpaired spouses. We investigated their *cardiovascular*-related health status including waist circumference, body mass index (BMI), blood pressure, and plasma lipids and compared results among centenarians, centenarian offspring, and offspring spouses.

**Results:**

Centenarians ranged from 99 to 109 years with a median age of 100 years. Centenarian offspring, with a median age of 70 years, and offspring spouses, with a median age of 69 years, shared similar age. Results of blood pressure, plasma lipid levels, and BMI displayed no significant difference between centenarian offspring and offspring spouses. However, centenarians appeared to have lower waist circumference, BMI, TC, LDL-C, TG, and diastolic blood pressure but higher levels of systolic blood pressure (*p* < 0.05). Multivariate analysis showed the prevalence of obesity, hypertension, and dyslipidemia was similar between centenarian offspring and offspring spouses, while centenarians appeared to have a lower prevalence of obesity and a higher prevalence of hypertension (*p* < 0.05).

**Conclusions:**

Centenarians and centenarian offspring did not present healthier BMI, blood pressure, or plasma lipids than offspring spouses. Further research on longevity and cardiovascular diseases are desirable.

## 1. Introduction

Cardiovascular diseases (CVDs) are the leading cause of disease burden and deaths in the world [1]. Due to population growth and aging, the burden of CVDs is increasing substantially in most low- and middle-income countries [1]. It is desirable to determine the underlying mechanisms for the development of feasible CVD prevention and to promote the ideal cardiovascular status and healthy aging [2].

Dyslipidemia, hypertension, diabetes, abdominal obesity, smoking, intake of fruits, vegetables, and alcohol, physical activity, and psychosocial factors account for over 90% of population attributable risks of myocardial infarction in the world [3]. The American Heart Association defined ideal health behaviors and factors including nonsmoking, adequate physical activity, healthy diet, normal body mass index (BMI), blood pressure, total cholesterol (TC), and fasting blood glucose [4]. Cardiovascular health-related factors, such as BMI, blood pressure, and lipids are associated with a variety of factors such as genes, age, diet, physical activity, and geographic environment [5–10]. Host genetics appear to influence personal responsiveness to dietary fats and cholesterol, and some risk alleles are found associated with hypertension, dyslipidemia, and obesity [6, 7, 9, 11]. The prevalence of vascular diseases dramatically increases with age [12]. Total cholesterol and triglyceride (TG) concentrations increase between 20 and 80 years of age but decrease in centenarians [13]. On the other hand, the contribution by environmental factors are increasingly recognized [1, 14, 15]: environmental exposures such as ambient air pollution, noise pollution, psychosocial, and economic stressors trigger risks for CVDs through various pathways [15]. Besides, familial environments affecting health-related behaviors play an important role. Hypertension, dyslipidemia, and obesity can be prevented by consuming a healthy diet including a limited intake of salt with adequate fruits and vegetables and engaging in healthy behaviors such as adequate physical activity and avoiding excessive alcohol intake [16–18].

Longevity and resistance to diseases such as CVDs may be regulated by shared underlying mechanisms [5]. There is genetic overlap between longevity and CVDs, where among 300 genes related to longevity, 87 are associated with cardiovascular-related traits including hypertension, lipid metabolism, energy metabolism, inflammation, and coagulation [5, 19–23]. Currently, there are many studies on centenarians and their offspring due to their perception as informative models for both longevity and CVDs. Studies revealed that centenarians and their offspring avoided or postponed the onset and progression of major CVDs [5, 24–26]. As for major cardiovascular-related factors such as BMI, blood pressure, and lipids, centenarians and their offspring seem to have genetic advantages with few risk variants and more protective variants [19, 21, 23, 27–29]. However, from the perspective of clinical phenotype, studies presented inconsistent results. Centenarians may have a lower prevalence of hypertension, but lower blood pressures are associated with poor cognitive function [30]. Lipids of centenarians from diverse geographical regions have been investigated, and inconsistent results showed that plasma levels of total cholesterol, low-density lipoprotein cholesterol (LDL-C), high-density lipoprotein cholesterol (HDL-C), and TG were either normal, lower, or not different than elderly controls [31–33]. Lower BMI was observed among centenarians, suggested as one of the keys for longevity, and may be due to their eating habits including low consumption of red meat and cholesterol [34–36]. With respect to centenarian offspring, they presented a lower prevalence of hypertension, dyslipidemia, and obesity in some studies, while other studies found no significant difference [37–41]. Environmental factors affect gene expression and may contribute to the heterogeneity of cardiovascular-related profiles among centenarians and their offspring [5, 42]. However, most studies were case-controls comprising of either centenarians and younger controls or centenarian offspring and age-matched controls; there are few studies investigating the health status of centenarian families including centenarians, centenarian offspring, and offspring spouses within the same environment.

As centenarians and their offspring may have genetic advantages, we hypothesized this population may present with healthier cardiovascular profiles. Therefore, we proposed to objectively determine cardiovascular health using cardiovascular-related health factors among recruited centenarians, their offspring, and offspring spouses without centenarian parents residing within the same environment to minimize the influence of environmental factors and better determine the extent of genetic components on CVD development and prevalence.

## 2. Study Design and Participants

The investigation was launched in Rugao, a county-level city in Jiangsu province in southern China. Rugao is renowned as a longevity city with the largest number of centenarians among county-level cities in China [43]. According to the data from the local Civil Affairs Bureau, Rugao had 394 centenarians in 2019.

Data were collected through a cross-sectional household-based survey of centenarian families. The ages of centenarians were verified according to their birth dates from identification cards. The study protocol was approved by the Ethical Committee of the Affiliated Hospital of Nantong University. The survey began in 2020 with the recruiting of centenarian families. We enrolled centenarians, centenarian offspring, and offspring spouses without centenarian parents. By May 6th, 2021, there were 253 centenarians, 217 centenarian offspring, and 116 offspring spouses who signed informed consents and cooperated with a questionnaire interview, physical health examination, and fasting blood sample collection. Among offspring and offspring spouses, there were 101 paired offspring and offspring spouses who lived together, 116 unpaired offspring, and 16 unpaired spouses.

## 3. Data Collection

Age was calculated from the date of birth recorded on identification cards. Health-related behaviors including smoking and alcohol use were collected using a structured questionnaire. Drug use was self-reported. Body mass was measured using an electronic scale and recorded in kilograms. Height and waist circumference were obtained using a flexible tape measure. Waist circumference was measured at the level of the navel. BMI was calculated as mass divided by height squared (kilograms/meters^2^). Right-arm blood pressure was checked using a standard electronic arm sphygmomanometer (1 mmhg = 0.133 kPa) under calm conditions. Fasting blood samples were collected by an experienced nurse and placed in a cold box at 4°C, before transport within 24 hours to the Affiliated Hospital of Nantong University's biobank. Plasma TC, LDL-C, HDL-C, and TG concentrations were assayed using an automated biochemical analyzer.

## 4. Definitions

Smoking status was classified as never smoker, former smoker, or current smoker. Alcohol status was classified as never drinker, former drinker, and current drinker. Using BMI, we classified participants as underweight (BMI <18.5 kg/m^2^), normal (BMI: 18.5–22.9 kg/m^2^), overweight (BMI: 23.0–27.4 kg/m^2^), or obese (BMI ≥27.5 kg/m^2^) [44]. Central obesity was defined by male waist circumference >90 cm or female waist circumference >85 cm [45]. Hypertension was defined by systolic blood pressure (SBP) ≥ 140 mmHg or diastolic blood pressure (DBP) ≥ 90 mmHg or under hypotensive therapy [46]. According to 2016 Chinese guidelines for managing dyslipidemia in adults, dyslipidemia was identified by meeting one or more of the following criteria: (1) high TC: TC ≥ 6.22 mmol/L (240 mg/dL), (2) high TG: TG ≥ 2.26 mmol/L (200 mg/dL), (3) High LDL-C: LDL-C ≥ 4.14 mmol/L (160 mg/dL), (4) Low HDL-C: HDL-C < 1.04 mmol/L (40 mg/dL), and (5) under lipid-lowering therapy [47, 48].

## 5. Statistical Analysis

Continuous variables were presented as medians with 25th and 75th percentiles due to skewed distribution. Categorical variables were presented as numbers and proportions. For continuous variables, we performed Kruskal–Wallis tests for comparison of three groups and nonparametric Mann–Whitney *U* tests for comparison of two groups. Categorical variables were examined using the chi-square test or Fisher's exact test. Bonferroni correction was used for multiple comparisons. We performed multivariate logistic regression on the prevalence of cardiovascular-related factors between groups. The regression model was adjusted for the effects of gender, smoking status, alcohol drinking status, and BMI. The odds ratio (OR) with 95% confidence intervals was used to measure association. *p* < 0.05 were considered statistically significant. All analyses were undertaken using SPSS 23.0 for windows.

## 6. Results

### 6.1. Demographics, Health-Related Behaviors, Physical Measures, Plasma Lipids, and Drug Use in Centenarian Families

Centenarians had a median age of 100 years, ranging from 99 to 109 years. Centenarian offspring, with a median age of 70 years, and offspring spouses, with a median age of 69 years, were mostly aged beyond 60 years and similar in age. Merely 26.1% of centenarians were males. Due to Chinese traditional culture, elderly parents prefer to live with their sons rather than their daughters. Thus, there were significantly more males (70.5%) in the group of centenarian offspring and more females (75.0%) in the group of offspring spouses (Table 1).

For health-related behaviors, both centenarians (15.6%) and centenarian offspring (19.1%) had more former smokers than offspring spouses (5.2%). Meanwhile, centenarian offspring (25.1%) had more current smokers than the other two groups. There were more current drinkers among centenarian offspring (48.6%) than centenarians (25.7%) and offspring spouses (28.1%). Centenarians (14.5%) had more former drinkers than offspring spouses (5.3%) (Table 1).

Centenarians displayed lower waist circumference, BMI, and diastolic blood pressure but higher levels of systolic blood pressure than centenarian offspring and offspring spouses (*p* < 0.05). No significant difference in these features was evidenced between centenarian offspring and offspring spouses (Table 1).

Regarding lipid plasma levels, centenarians showed significantly lower levels of TC, LDL-C, and TG than centenarian offspring and offspring spouses (*p* < 0.05). When compared to centenarian offspring, centenarians had low levels of HDL-C (*p* < 0.05). No significant difference in plasma lipids was observed between centenarian offspring and offspring spouses (Table 1).

Regarding the current pharmacologic therapy, a lower percentage (19.0%) of centenarians underwent hypotensive therapy than centenarian offspring (30.4%) and offspring spouses (37.9%). Centenarians (0.0%) were treated less with lipid-lowering drugs than centenarian offspring (3.7%). Centenarian offspring and offspring spouses shared similar results on hypotensive and lipid-lowering drug use (Table 1).

### 6.2. Prevalence of Cardiovascular-Related Factors between Centenarian, Centenarian Offspring, and Offspring Spouses

Since the numbers of males and that of females among the three groups are not equal, we compared the prevalence of cardiovascular-related factors among male and female participants. Female centenarians were more likely to have high TC (*p*=0.005) than males. No significant difference in prevalence for hypertension, overweight, obesity, central obesity, high LDL-C, low HDL-C, high TG, and dyslipidemia among male and female centenarians was noted (Table 2). Among centenarian offspring, females had a higher prevalence of high TC, high LDL, high TG, and dyslipidemia than males (*p* < 0.05) (Supplementary Table 1). Among offspring spouses, males had a higher prevalence of low HDL than females (*p* < 0.05) (Supplementary Table 2). We did not find a significant difference in other cardiovascular-related factors among male and female participants.

In total, 72.2% of centenarians suffered hypertension, 9.6% suffered obesity, 37.7% suffered central obesity, and 29.2% suffered dyslipidemia. Low levels of HDL-C were the most common type of dyslipidemia among centenarians (19.4%). Then, we considered offspring spouses as the control group. After adjusting for possible confounders such as gender, smoking status, and alcohol drinking status, centenarians had reduced prevalence of overweight (OR 0.336, 95% CI 0.197–0.572, *p* < 0.001), obesity (OR 0.245, 95% CI 0.125–0.480, *p* < 0.001), central obesity (OR 0.466, 95% CI 0.285–0.762, *p*=0.002), high TC (OR 0.421, 95% CI 0.193–0.919, *p*=0.030), high LDL-C (OR 0.450, 95% CI 0.209–0.969, *p*=0.041), high TG (OR 0.198, 95% CI 0.077–0.511, *p* < 0.001) and greater prevalence of hypertension (OR 2.589, 95% CI 1.426–4.699, *p*=0.002). The difference in prevalence for dyslipidemia was not significant (Table 3).

However, centenarian offspring and offspring spouses shared similar risks of hypertension, overweight, obesity, central obesity, high TC, high LDL-C, low HDL-C, high TG, and dyslipidemia (Table 4). Furthermore, comparison between subgroups, including paired offspring and offspring spouses who lived together, unpaired offspring, and unpaired spouses, found no significant difference in cardiovascular-related factors (Supplementary Table 3).

## 7. Discussion

Centenarians are recognized as the product of their personal genetics × environment equation and human longevity clusters in families [5]. However, few studies focus on centenarian families where centenarians, centenarian offspring, and offspring spouses live in the same environment. In our study, we recruited centenarian families in Rugao and controlled for the geographic and cultural environmental difference. Furthermore, we enrolled paired centenarian offspring and offspring spouses within the same familial environment.

We found that centenarians had lower prevalence of overweight, obesity, central obesity, high TC, high LDL-C, and high TG than elderly controls, as previously reported in Hainan and Portuguese centenarians [13, 36]. Also, decreased TC, LDL-C, and TG concentrations were considered as possibly beneficial factors preventing centenarians from cardiovascular diseases and promoting longevity [13]. However, centenarian offspring did not present a similar profile as their centenarian parents. In our study, they shared similar risks of hypertension, obesity, central obesity, and dyslipidemia with offspring spouses. In the absence of data on venous blood glucose, the prevalence of diabetes was not analyzed in our study. A previous study on Italian centenarian offspring reported a similar prevalence of diabetes between centenarian offspring and controls of non-long-lived parents, while American and Ashkenazi Jewish centenarian offspring were reported with a lower prevalence of diabetes [26, 37, 49]. The discrepancy was thought to be associated with the environmental context, cultural habits, and lifestyle factors [37].

In our study, female centenarians had a greater prevalence of high TC than males, and sex differentials on lipid profiles were also discovered among centenarian offspring and offspring spouses. In general population, male is a risk factor for dyslipidemia [47]. But with the increase of age, the sex difference on lipid concentrations narrows, and menopause contributes to increased levels of TC, LDL-C, and TG, and decreased levels of HDL-C in women is associated with reduced oestrogen, which may lead to higher TC levels in female centenarians [47, 50, 51]. Besides, centenarians and centenarian offspring appeared to have larger percentages of smokers and alcohol drinkers than offspring spouses. Also, the effects of cigarette smoking and alcohol drinking on CVDs have been discovered in previous studies [52, 53]. Thus, we performed multivariate analysis on the prevalence of cardiovascular-related factors adjusting for the effects of gender, smoking status, and alcohol drinking status.

Cardiovascular-related factors such as BMI, blood pressure, and lipids are influenced by multiple factors. Although we recruited participants residing in the same geographic, cultural, and familial area, they may adopt different diets and behaviors, which substantially affect risks of obesity, hypertension, and dyslipidemia [16–18]. Obesity is an independent risk factor for cardiovascular diseases [10]. In our study, centenarian offspring and offspring spouses shared similar BMI and prevalence of overweight and obesity. Our finding may reflect a weaker genetic effect and a stronger environmental effect on BMI. Twin and adoption studies revealed a 50%–80% of heritability on obesity [54, 55]. However, genetic studies demonstrate a weak explanation by genetic variance [56]. A longitudinal study in Norway implies environment is the main contributor to BMI [55]. Centenarians had lower waist circumference, BMI, and prevalence of overweight and obesity, corresponding to other research on centenarians [35, 36, 57]. However, these features may result from lower energy intake and frailty [34, 35].

Higher blood pressure and LDL-C contribute to adverse cardiometabolic events among elderly people [58]. In our study, we did not find a significant difference between centenarian offspring and offspring spouses regarding blood pressure and plasma lipids, in contrast with studies reporting a lower prevalence of hypertension and hypercholesterolemia among centenarian offspring than age-matched controls [37, 39, 40]. Similar research on centenarian offspring which recruited spouses as controls found no significant difference in the prevalence of hypertension and new onset of hypertension during follow-up [25]. These findings may indicate a greater impact of environment on hypertension than genetic factors. The effect of environmental factors may also contribute to lipid result interpretation [38, 47, 59]. However, future studies are needed to clarify the differential results across studies. Centenarians had a higher prevalence of hypertension, which corresponds with previous studies revealing the high prevalence of age-related diseases in centenarians such as hypertension, stroke, and heart disease [35, 60]. As for lipids, centenarians presented lower levels of TC, LDL-C, and TG. Whether this lipid profile among centenarians is a predictor of exceptional longevity or it is a consequence of aging is still unclear [31, 33, 57]. No significant difference was found concerning HDL-C levels between centenarians and offspring spouses. HDL-C was strongly associated with genetic factors and generally decreases with age [61, 62]. Additionally, HDL-C has been considered as a more important and protective factor in elderly people [61]. Thus, the reserve of HDL-C levels in centenarians may be associated with the longevity phenotype.

However, there are several limitations in this study. First, our cross-sectional study is based on centenarian families in Rugao, which fails to represent all centenarian families, and longitudinal studies are needed to provide stronger evidence. Second, possible confounders such as diet, physical activity, sleep condition, and other cardiovascular-related factors such as fasting blood glucose and history of CVDs were not analyzed. Finally, further studies are needed to determine genetic and environmental effects on longevity and CVDs.

## 8. Conclusions

In conclusion, centenarians and centenarian offspring did not present significantly improved blood pressure, plasma lipids, or BMI in comparison with offspring spouses. Further research on longevity and cardiovascular diseases are recommended to elucidate contributing factors to CVD development and cultivate successful intervention strategies.

## Figures and Tables

**Table 1:** Demographics, health-related behaviors, physical measures, plasma lipids, and drug use in centenarian families.

	Centenarian *n* = 253	Centenarian offspring *n* = 217	Offspring spouses *n* = 116	*p* value
Demographics				
Age	100.0 (99.0–101.0)^#^	70.0 (65.0–75.0)^*∗*^	69.0 (64.5–74.0)	<0.001
Male, *n* (%)	66 (26.1%)	153 (70.5%)^#^^*∗*^	29 (25.0%)	<0.001
Smoking status				
Current smoker, *n* (%)	12 (4.8%)	54 (25.1%)^#^^*∗*^	12 (10.4%)	<0.001
Former smoker, *n* (%)	39 (15.6%)^#^	41 (19.1%)^#^	6 (5.2%)	0.003
Alcohol drinking status				
Current drinker, *n* (%)	64 (25.7%)	104 (48.6%)^#^^*∗*^	32 (28.1%)	<0.001
Former drinker, *n* (%)	36 (14.5%)^#^	16 (7.5%)	6 (5.3%)	0.007
Physical measures				
Waist circumference (cm)	84.0 (76.0–90.0)^#^	87.0 (80.0–93.0)^*∗*^	88.0 (81.0–97.0)	0.001
BMI (kg/m^2^)	21.12 (18.79–23.73)^#^	24.25 (22.29–26.58)^*∗*^	24.90 (22.26–27.75)	<0.001
SBP (mmHg)	150 (132–169)^#^	141 (126–157)^*∗*^	140 (124–159)	<0.001
DBP (mmHg)	72 (64–82)^#^	80 (73–87)^*∗*^	77 (71–85)	<0.001
Lipids				
TC (mmol/L)	4.6 (4.1–5.3)^#^	5.1 (4.5–5.8)^*∗*^	5.2 (4.5–5.8)	<0.001
LDL-C (mmol/L)	2.89 (2.47–3.35)^#^	3.26 (2.77–3.78)^*∗*^	3.34 (2.85–3.84)	<0.001
HDL-C (mmol/L)	1.32 (1.09–1.53)	1.36 (1.20–1.60)^*∗*^	1.36 (1.13–1.55)	0.049
TG (mmol/L)	1.02 (0.85–1.37)^#^	1.15 (0.86–1.63)^*∗*^	1.24 (0.87–1.87)	0.002
Drug use				
Hypotensive therapy, *n* (%)	48 (19.0%)^#^	66 (30.4%)^*∗*^	44 (37.9%)	<0.001
Lipid-lowering therapy, *n* (%)	0 (0.0%)	8 (3.7%)^*∗*^	1 (0.9%)	0.002

*n*, number; %, percentage; BMI, body mass index; SBP, systolic blood pressure; DBP, diastolic blood pressure; TC, total cholesterol; LDL-C, low-density lipoprotein cholesterol; HDL-C, high-density lipoprotein cholesterol; TG, triglycerides. ^#^Bonferroni-corrected *p* < 0.05 versus offspring spouses. ^*∗*^Bonferroni-corrected *p* < 0.05 versus centenarians.

**Table 2:** Prevalence of cardiovascular-related factors in centenarians stratified by gender.

	Male	Female	*p* value
Hypertension, *n* (%)	43 (68.3%)	131 (73.6%)	0.416
Overweight, *n* (%)	17 (28.3%)	26 (17.7%)	0.087
Obesity, *n* (%)	9 (15.0%)	11 (7.4%)	0.093
Central obesity, *n* (%)	19 (32.8%)	56 (39.7%)	0.357
High TC, *n* (%)^*∗*^	0 (0.0%)	19 (10.2%)	0.005
High LDL-C, *n* (%)^*∗*^	2 (3.0%)	18 (9.6%)	0.088
Low HDL-C, *n* (%)^*∗*^	17 (25.8%)	32 (17.1%)	0.127
High TG, *n* (%)^*∗*^	3 (4.5%)	8 (4.3%)	1.000
Dyslipidemia, *n* (%)	19 (28.8%)	55 (29.4%)	0.924

*n*, number; %, percentage; TC, total cholesterol; LDL-C, low-density lipoprotein cholesterol; HDL-C, high-density lipoprotein cholesterol; TG, triglycerides. ^*∗*^Subjects under lipid-lowering agents were excluded from the analysis.

**Table 3:** Prevalence of cardiovascular-related factors between centenarians and offspring spouses.

	Centenarian	Offspring spouses	OR (95% CI)	*p* value
Hypertension, *n* (%)^a^	174 (72.2%)	67 (59.8%)	2.589 (1.426–4.699)	0.002
Overweight, *n* (%)^b^	43 (20.8%)	47 (42.3%)	0.336 (0.197–0.572)	<0.001
Obesity, *n* (%)^b^	20 (9.6%)	31 (27.7%)	0.245 (0.125–0.480)	<0.001
Central obesity, *n* (%)^b^	75 (37.7%)	62 (55.9%)	0.466 (0.285–0.762)	0.002
High TC, *n* (%)^*∗*^^a^	19 (7.5%)	21 (18.3%)	0.421 (0.193–0.919)	0.030
High LDL-C, *n* (%)^*∗*^^a^	20 (7.9%)	21 (18.3%)	0.450 (0.209–0.969)	0.041
Low HDL-C, *n* (%)^*∗*^^a^	49 (19.4%)	10 (8.7%)	2.629 (1.140–6.063)	0.023
High TG, *n* (%)^*∗*^^a^	11 (4.3%)	22 (19.1%)	0.198 (0.077–0.511)	<0.001
Dyslipidemia, *n* (%)^a^	74 (29.2%)	39 (33.6%)	0.885 (0.508–1.539)	0.664

*n*, number; %, percentage; OR, odds ratio; CI, confidence interval; TC, total cholesterol; LDL-C, low-density lipoprotein cholesterol; HDL-C, high-density lipoprotein cholesterol; TG, triglycerides. ^*∗*^Subjects under lipid-lowering agents were excluded from the analysis. ^a^Adjusted for gender, smoking status, alcohol drinking status, and BMI. ^b^Adjusted for gender, smoking status, and alcohol drinking status.

**Table 4:** Prevalence of cardiovascular-related factors between centenarian offspring and offspring spouses.

	Centenarian offspring	Offspring spouses	OR (95% CI)	*p* value
Hypertension, *n* (%)^a^	133 (62.1%)	67 (59.8%)	1.343 (0.763–2.364)	0.307
Overweight, *n* (%)^b^	103 (49.8%)	47 (42.3%)	1.380 (0.811–2.348)	0.235
Obesity, *n* (%)^b^	37 (17.8%)	31 (27.7%)	0.575 (0.305–1.086)	0.088
Central obesity, *n* (%)^b^	89 (42.4%)	62 (55.9%)	0.600 (0.354–1.016)	0.057
High TC, *n* (%)^*∗*^^a^	28 (13.4%)	21 (18.3%)	1.291 (0.634–2.628)	0.481
High LDL-C, *n* (%)^*∗*^^a^	30 (14.4%)	21 (18.3%)	1.208 (0.597–2.443)	0.600
Low HDL-C, *n* (%)^*∗*^^a^	21 (10.0%)	10 (8.7%)	1.188 (0.450–3.138)	0.728
High TG, *n* (%)^*∗*^^a^	24 (11.5%)	22 (19.1%)	0.991 (0.477–2.059)	0.982
Dyslipidemia, *n* (%)^a^	65 (30.0%)	39 (33.6%)	1.243 (0.699–2.212)	0.459

*n*, number; %, percentage; OR, odds ratio; CI, confidence interval; TC, total cholesterol; LDL-C, low-density lipoprotein cholesterol; HDL-C, high-density lipoprotein cholesterol; TG, triglycerides. ^*∗*^Subjects under lipid-lowering agents were excluded from the analysis. ^a^Adjusted for gender, smoking status, alcohol drinking status, and BMI. ^b^Adjusted for gender, smoking status, and alcohol drinking status.

## Data Availability

The raw data required to reproduce these findings cannot be shared at this time as the data also forms part of an ongoing study.

## Supplementary Materials

Supplementary Materials Supplementary Table 1: prevalence of cardiovascular-related factors in centenarian offspring stratified by gender. Supplementary Table 2: prevalence of cardiovascular-related factors in offspring spouses stratified by gender. Supplementary Table 3: prevalence of cardiovascular-related factors between subgroups.
