# Supplementary material for: BMI, Blood Pressure, and Plasma Lipids among Centenarians and Their Offspring
Source: Evid Based Complement Alternat Med. 2022 Jan 20;2022:3836247. doi: 10.1155/2022/3836247 (PMC8794670; doi:10.1155/2022/3836247)
Supplement: Supplementary Materials — Supplementary Table 1: prevalence of cardiovascular-related factors in centenarian offspring stratified by gender. Supplementary Table 2: prevalence of cardiovascular-related factors in offspring spouses stratified by gender. Supplementary Table 3: prevalence of cardiovascular-related factors between subgroups. [file 3836247.f1.docx]

Supplementary Table 1 Prevalence of cardiovascular-related factors in centenarian offspring stratified by gender

|  | Male | Female | *P* value |
| --- | --- | --- | --- |
| Hypertension, n (%) | 93(61.6%) | 40(63.5%) | 0.794 |
| Overweight, n (%) | 74(50.7%) | 29(47.5%) | 0.680 |
| Obesity, n (%) | 23(15.6%) | 14(23.0%) | 0.210 |
| Central Obesity, n (%) | 61(41.2%) | 28(45.2%) | 0.598 |
| High TC, n(%)^*^ | 8(5.5%) | 20(31.7%) | ＜0.001 |
| High LDL-C, n(%)^*^ | 10(6.8%) | 20(31.7%) | ＜0.001 |
| Low HDL-C, n(%)^*^ | 16(11.0%) | 5(7.9%) | 0.505 |
| High TG, n(%)^*^ | 11(7.5%) | 13(20.6%) | 0.006 |
| Dyslipidemia, n(%) | 36(23.5%) | 29(45.3%) | 0.001 |

Abbreviations: n, number; %, percentage; TC, total cholesterol; LDL-C, low-density lipoprotein cholesterol; HDL-C, high-density lipoprotein cholesterol; TG, triglycerides.

*: Subjects under Lipid-lowering agents were excluded from the analysis.

Supplementary Table 2 Prevalence of cardiovascular-related factors in offspring spouses stratified by gender

|  | Male | Female | *P* value |
| --- | --- | --- | --- |
| Hypertension, n (%) | 17(60.7%) | 50(59.5%) | 0.911 |
| Overweight, n (%) | 11(39.3%) | 36(43.4%) | 0.705 |
| Obesity, n (%) | 10(35.7%) | 21(25.0%) | 0.272 |
| Central Obesity, n (%) | 14(48.3%) | 48(58.5%) | 0.339 |
| High TC, n(%)^*^ | 5(17.2%) | 16(18.6%) | 0.869 |
| High LDL-C, n(%)^*^ | 7(24.1%) | 14(16.3%) | 0.343 |
| Low HDL-C, n(%)^*^ | 6(20.7%) | 4(4.7%) | 0.016 |
| High TG, n(%)^*^ | 6(20.7%) | 16(18.6%) | 0.805 |
| Dyslipidemia, n(%) | 12(41.4%) | 27(31.0%) | 0.307 |

Abbreviations: n, number; %, percentage; TC, total cholesterol; LDL-C, low-density lipoprotein cholesterol; HDL-C, high-density lipoprotein cholesterol; TG, triglycerides.

*: Subjects under Lipid-lowering agents were excluded from the analysis.

Supplementary Table 3 Prevalence of cardiovascular-related factors between subgroups

|  | Offspring  N=101 | Spouses  N=101 | Unpaired Offspring  N=116 | Unpaired Spouses  N=15 | *P* value |
| --- | --- | --- | --- | --- | --- |
| Hypertension, n (%) | 57(57.6%) | 57(58.8%) | 76(66.1%) | 10(66.7%) | 0.545 |
| Overweight, n (%) | 50(51.5%) | 45(45.9%) | 53(48.2%) | 2(15.4%) | 0.106 |
| Obesity, n (%) | 18(18.4%) | 25(25.5%) | 19(17.3%) | 6(42.9%) | 0.097 |
| Central Obesity, n (%) | 41(41.8%) | 53(54.6%) | 48(42.9%) | 9(64.3%) | 0.123 |
| High TC, n(%)^*^ | 15(15.0%) | 17(17.0%) | 13(11.9%) | 4(26.7%) | 0.407 |
| High LDL-C, n(%)^*^ | 14(14.0%) | 17(17.0%) | 16(14.7%) | 4(26.7%) | 0.575 |
| Low HDL-C, n(%)^*^ | 8(8.0%) | 8(8.0%) | 13(11.9%) | 2(13.3%) | 0.625 |
| High TG, n(%)^*^ | 13(13.0%) | 19(19.0%) | 11(10.1%) | 3(20.0%) | 0.238 |
| Dyslipidemia, n(%) | 28(27.7%) | 33(32.7%) | 37(31.9%) | 6(40.0%) | 0.728 |

Abbreviations: n, number; %, percentage; TC, total cholesterol; LDL-C, low-density lipoprotein cholesterol; HDL-C, high-density lipoprotein cholesterol; TG, triglycerides.

*: Subjects under Lipid-lowering agents were excluded from the analysis.
